# Supplementary material for: Contemporary Research Progress on the Detection of Polycyclic Aromatic Hydrocarbons
Source: Int J Environ Res Public Health. 2022 Feb 27;19(5):2790. doi: 10.3390/ijerph19052790 (PMC8910359; doi:10.3390/ijerph19052790)
Supplement: Supplementary file 1 [file ijerph-19-02790-s001.zip › Table S1.pdf]

Table S1. Some vibration modes of fluoranthene and pyrene.

**fluoranthene**			**pyrene**		
Frequency/cm^{-1}	Infrared/cm^{-1}	Vibration Mode	Frequency/cm^{-1}	Infrared/cm^{-1}	Vibration Mode
150.12	124.3243	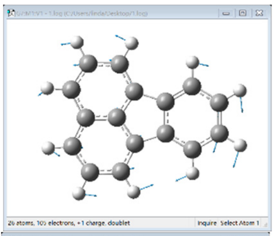	873.98	104.9775	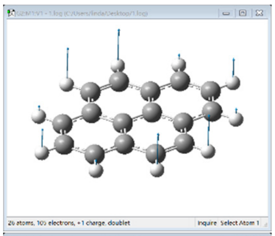
331.98	122.5875	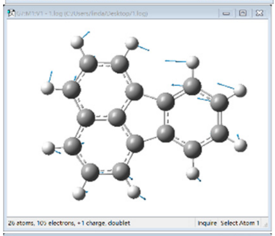	1266.45	77.1621	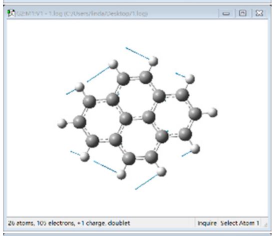
722.61	221.675	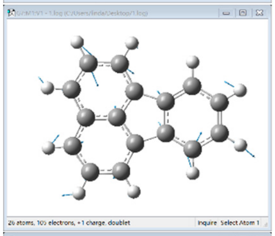	1373.23	139.1556	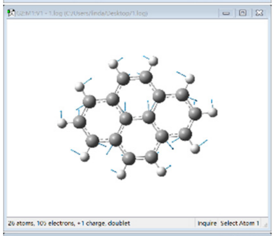
947.12	633.6108	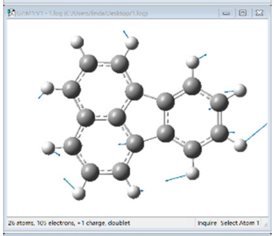	1580.05	176.99	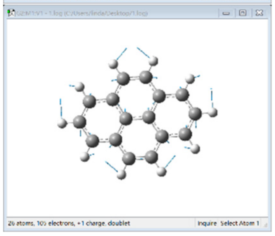
1300	142.1205	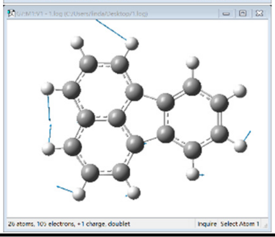			
